# Renal Association Clinical Practice Guideline on peritoneal dialysis in adults and children

**DOI:** 10.1186/s12882-017-0687-2

**Published:** 2017-11-16

**Authors:** Graham Woodrow, Stanley L. Fan, Christopher Reid, Jeannette Denning, Andrew Neil Pyrah

**Affiliations:** 1 St James’s University Hospital Leeds Teaching Hospitals NHS Trust, Leeds, UK; 2 Royal London Hospital, London, UK; 3 Evelina Children’s Hospital, Guy’s and St Thomas’ NHS Trust, London, UK; 4 Peritoneal Dialysis Unit, St James’s University Hospital Leeds Teaching Hospitals NHS Trust, London, UK; 5 Patient Representative, c/o The Renal Association, Bristol, UK

## Abstract

These guidelines cover all aspects of the care of patients who are treated with peritoneal dialysis. This includes equipment and resources, preparation for peritoneal dialysis, and adequacy of dialysis (both in terms of removing waste products and fluid), preventing and treating infections. There is also a section on diagnosis and treatment of encapsulating peritoneal sclerosis, a rare but serious complication of peritoneal dialysis where fibrotic (scar) tissue forms around the intestine. The guidelines include recommendations for infants and children, for whom peritoneal dialysis is recommended over haemodialysis.

Immediately after the introduction there is a statement of all the recommendations. These recommendations are written in a language that we think should be understandable by many patients, relatives, carers and other interested people. Consequently we have not reworded or restated them in this lay summary. They are graded 1 or 2 depending on the strength of the recommendation by the authors, and A-D depending on the quality of the evidence that the recommendation is based on.

## Introduction

These guidelines cover the organisation and performance of peritoneal dialysis as a treatment for kidney patients, including infants and children. It includes prevention and treatment of complications. It does not include factors involved in the choice of peritoneal dialysis compared to other options for patients with stage 5 chronic kidney disease. This document is intended for use by any member of the health care team engaged in the care of kidney patients treated with peritoneal dialysis.

Peritoneal dialysis (PD) is long established as a major option for renal replacement therapy in patients with end-stage renal disease. It is an important part of an integrated service for renal replacement therapy that is frequently selected by patients as their preferred initial mode of therapy and is a therapeutic option for patients wishing or needing to swap from HD and after renal transplant failure. PD is the best option for infants and small children. NICE Clinical Guideline 125 (2011) recommends PD as the initial dialysis treatment of choice of chronic kidney disease stage 5 for children aged 2 years or older, people with residual renal function and adults without significant associated comorbidities.

For the first time, this Renal Association guideline includes recommendations relating to PD in children. Recommendations in this guideline will refer to both adult and paediatric patients, except where the recommendation specifies one of these patient groups or provides alternative recommendations to them.

This guideline is an update of the PD module published on-line on the Renal Association website, www.renal.org in 2010. The English language literature was searched in December 2016 to identify relevant articles on PD published between 2008 and 2016 including:

- Medline search using ‘peritoneal dialysis’ combined with relevant terms from each of the sections - Equipment & Resources, Training & Catheter Insertion, Dialysis Clearance, Ultrafiltration & Overhydration, Infections, Peritonitis, Exit Site Infections, Renal Osteodystrophy & Diabetes Mellitus, Encapsulating Peritoneal Sclerosis, Assisted Peritoneal Dialysis, Icodextrin, Peritoneal Membrane, Urgent Start and Biocompatible Solutions
- Cochrane Database of Systematic Reviews
- Review of other national/international PD clinical guidelines
- Identification of further articles quoted in identified papers
- Review of Peritoneal Dialysis International’s table of contents for articles relating to the content of the guidelines
- Searches within the major renal journals (Journal of the American Society of Nephrology, Clinical Journal of the American Society of Nephrology, Nephrology Dialysis Transplantation, Kidney International, American Journal of Kidney Diseases) for articles with ‘peritoneal dialysis’ in the title/abstract

The recommendations in this guideline have been harmonised with other PD guidelines whenever possible and the recommendations to follow international PD or other Renal Association guidelines have not been graded.

## Summary of clinical practice guidelines for peritoneal dialysis

### Peritoneal dialysis (PD) (guidelines PD 1.1–1.5)

#### Guideline 1.1.1 – PD: Equipment and resources

We recommend that Peritoneal Dialysis should be delivered in the context of a comprehensive and integrated service for renal replacement therapies, including haemodialysis (including temporary backup facilities), transplantation and conservative care. Both continuous ambulatory peritoneal dialysis (CAPD) and automated peritoneal dialysis (APD), in all its forms should be available (1C).

#### Guideline 1.1.2 – PD: Equipment and resources

We recommend that a dedicated PD nursing team should be part of the multidisciplinary team (1C).

#### Guideline 1.1.3 – PD: Equipment and resources

We recommend that where feasible, each unit has a designated lead clinician for PD (1C).

#### Guideline 1.1.4 – PD: Equipment and resources

We recommend that assisted PD should be available to patients wishing to have home dialysis treatment but unable to perform self-care PD, including as a temporary measure where a patient who is, or will become, independent is unable to perform PD alone (1C).

#### Guideline 1.2 – PD: Equipment and resources

We recommend that all equipment and fluid used in the delivery and monitoring of PD therapies should comply with the relevant standards for medical fluids and devices (1C).

#### Guideline 1.3 – PD: Equipment and resources

We recommend that the use of disconnect systems should be standard unless clinically contraindicated (1A).

#### Guideline 1.4 – PD: Equipment and resources

We suggest that biocompatible PD solutions (solutions that have normal pH and/or low concentrations of glucose degradation products) should be used in patients experiencing infusion pain (2B).

#### Guideline 1.5 – PD: Equipment and resources

We suggest that biocompatible PD solutions (normal pH and/or low concentrations of glucose degradation products) may be considered for better preservation of residual renal function with long term (>12 month) use (2B).

### Peritoneal dialysis (PD) (guidelines PD 2.1–2.4)

#### Guideline 2.1 – PD: Preparation for peritoneal dialysis

We recommend that all patients (and parents of paediatric patients) should, where possible, be adequately prepared for renal replacement therapy and this should include receiving information and education about PD treatment, delivered by an experienced member of the MDT. Patients commencing RRT in an unplanned fashion for whatever reason should receive this information once appropriate (1C). Fast track education and urgent PD catheter insertion with acute start of PD should be available, and be offered to suitable patients urgently starting on RRT who wish to avoid temporary haemodialysis (1C).

#### Guideline 2.2 – PD: Preparation for peritoneal dialysis

We recommend that, where possible, timing of PD catheter insertion should be planned to accommodate patient convenience, commencement of training between 10 days and 6 weeks and before RRT is essential to enable correction of early catheter-related problems without the need for temporary haemodialysis (1C).

#### Guideline 2.3 – PD: Preparation for peritoneal dialysis

We recommend that PD catheter insertion practice should be managed according to the Renal Association Peritoneal Access Guidelines. Paediatric PD access procedures will routinely be performed under general anaesthetic (Ungraded).

#### Guideline 2.4 – PD: Preparation for peritoneal dialysis

We recommend that peri-operative catheter care and catheter complications (leaks, hernias, obstruction) should be managed according to the International Society of Peritoneal Dialysis guidelines 2005, and for children, the European Elective Chronic Peritoneal Dialysis Guideline 2001 (Ungraded).

### Peritoneal dialysis (PD) (guidelines PD 3.1–3.3)

#### Guideline 3.1 – PD: Solute clearance

We recommend that both residual urine and peritoneal dialysis components of small solute clearance should be measured at least six monthly or more frequently if dependant on residual renal function to achieve clearance targets or if clinically or biochemically indicated in adults and in children. Both urea and/or creatinine clearances can be used to monitor dialysis adequacy and should be interpreted within the limits of the methods (1C).

#### Guideline 3.2.1 – PD: Solute clearance

We recommend that a combined urinary and peritoneal Kt/V_urea_ of 1.7/week or a creatinine clearance of 50 L/week/1.73m^2^ should be considered as minimal treatment doses for adults (1A). We recommend/suggest that clearance targets for children should be a minimum of those for adults (1C).

#### Guideline 3.2.2 – PD: Solute clearance

We recommend that the dose of dialysis should be increased in patients experiencing uraemic symptoms, or inadequate growth in children, even if meeting minimum clearance targets (1B).

#### Guideline 3.3 – PD: Solute clearance

We recommend that a continuous 24 h PD regime is preferred to an intermittent regime for anuric patients (1B).

### Peritoneal dialysis (PD) (guidelines PD 4.1–4.5)

#### Guideline 4.1 – PD: Ultrafiltration and fluid management

We recommend that peritoneal membrane function should be monitored regularly (6 weeks after commencing treatment and at least annually or when clinically indicated) using a peritoneal equilibration test (PET) or equivalent. Daily urine and peritoneal ultrafiltration volumes, with appropriate correction for overfill, should be monitored at least six-monthly (1C).

#### Guideline 4.2 – PD: Ultrafiltration and fluid management

We recommend that dialysis regimens resulting in fluid reabsorption should be avoided. Patients with high or high average solute transport, at greatest risk of this problem, should be considered for APD and icodextrin (1A).

#### Guideline 4.3 – PD: Ultrafiltration and fluid management

We recommend that dialysis regimens resulting in routine utilisation of hypertonic (3.86%) glucose exchanges should be minimised. Where appropriate this should be achieved by using icodextrin or diuretics (1B).

#### Guideline 4.4 – PD: Ultrafiltration and fluid management

We recommend that treatment strategies that favour preservation of renal function or volume should be adopted where possible. These include the use of ACEi, ARBs (in adults only) and diuretics, and the avoidance of episodes of dehydration (1B).

#### Guideline 4.5 – PD: Ultrafiltration and fluid management

We recommend that anuric patients who are overhydrated and consistently achieve a daily ultrafiltration of less than 750 ml in adults (or equivalent volume for body size in paediatrics) should be closely monitored. These patients may benefit from prescription changes and/or modality switch (1B).

### Peritoneal dialysis (PD) (guidelines PD 5.1–5.2)

#### Guideline 5.1 – PD: Infectious complications

##### Guideline 5.1.1 – PD infectious complications: Prevention strategies

We recommend that PD units should undertake regular audit of their peritonitis and exit-site infection rates, including causative organism, treatment and outcomes. They should enter into active dialogue with their microbiology department and infection control team to develop optimal local treatment and prevention protocols (1B).

##### Guideline 5.1.2 – PD infectious complications: Prevention strategies

We recommend that flush-before-fill dialysis delivery systems should be used for CAPD (1A).

##### Guideline 5.1.3 – PD infectious complications: Prevention strategies

We recommend that patients (and/or carers or parents) should undergo regular revision of their technique (at least annually or more frequently if indicated, such as after an episode of PD-related infection or a significant interruption to the patient performing PD) and receive intensified training if this is below standard (1C).

##### Guideline 5.1.4 – PD infectious complications: Prevention strategies

We recommend that initial catheter insertion should be accompanied by antibiotic prophylaxis (1B).

##### Guideline 5.1.5 – PD infectious complications: Prevention strategies

We recommend that invasive procedures should be accompanied by antibiotic prophylaxis and emptying the abdomen of dialysis fluid for a period commensurate with the procedure (1C).

##### Guideline 5.1.6 – PD infectious complications: Prevention strategies

We recommend that topical antibiotic administration should be used to reduce the frequency of exit-site infection and peritonitis (1A).

#### Guideline 5.2 – PD: Infectious complications

##### Guideline 5.2.1 – PD infectious complications: Treatment

We recommend that exit site infection is suggested by pain, swelling, crusting, erythema and serous discharge; purulent discharge always indicates infection. Swabs should be taken for culture and initial empiric therapy should be with oral antibiotics that will cover *S. aureus and P. aeruginosa* (1B).

##### Guideline 5.2.2 – PD infectious complications: Treatment

We recommend that methicillin resistant organisms (MRSA) will require systemic treatment (e.g. vancomycin) and will need to comply with local infection control policies (1C).

##### Guideline 5.2.3 – PD infectious complications: Treatment

We recommend that initial treatment regimens for peritonitis should include cover for bacterial Gram positive and Gram negative organisms including *Pseudomonas species* until result of culture and antibiotic sensitivities are obtained (1C).

### Peritoneal dialysis (PD) (guidelines PD 6.1–6.4)

#### Guideline 6.1 – PD: Metabolic factors

We recommend that standard strategies to optimise diabetic control should be used; these should be complemented by dialysis prescription regimens that minimise glucose, including glucose free-solutions (icodextrin and amino-acids), where possible (1B).

#### Guideline 6.2 – PD: Metabolic factors

We recommend that plasma bicarbonate should be maintained within the normal range. This can be achieved in the vast majority of patients by adjusting the dialysis dose and/or dialysate buffer concentration (1B).

#### Guideline 6.3 – PD: Metabolic factors

We suggest that central obesity can worsen or develop in some PD patients. The risk of this problem, and associated metabolic complications, notably increased atherogenicity of lipid profiles and insulin resistance, can be reduced by avoiding excessive glucose prescription and using icodextrin (2C).

#### Guideline 6.4 – PD: Metabolic factors

We recommend that awareness of the effects of icodextrin on assays for estimation of amylase and glucose (using glucose dehydrogenase) should be disseminated to patients, relatives, laboratory and clinical staff (1C).

### 7. Peritoneal dialysis (PD) (guidelines PD 7.1)

#### Guideline 7.1 – PD: Encapsulating peritoneal sclerosis

##### Guideline 7.1.1 – PD: Encapsulating peritoneal sclerosis: Diagnosis

We recommend that the diagnosis of encapsulating peritoneal sclerosis (EPS) requires the presence of a combination of clinical and radiological features of intestinal obstruction and encapsulation GRADE 1B.

##### Guideline 7.1.2 – PD: Encapsulating peritoneal sclerosis: Diagnosis

We recommend that the radiological technique of choice for the diagnosis of encapsulating peritoneal sclerosis (EPS) is CT scanning GRADE 1B.

##### Guideline 7.1.3 – PD: Encapsulating peritoneal sclerosis: Diagnosis

We recommend that radiological and biochemical screening methods are NOT of sufficient sensitivity and specificity to be used clinically to identify early or imminent development of EPS in asymptomatic PD patients (GRADE 1C).

#### Guideline 7.2 – PD: Encapsulating peritoneal sclerosis

##### Guideline 7.2.1 – PD: Encapsulating peritoneal sclerosis: Management

We recommend that patients with suspected encapsulating peritoneal sclerosis (EPS) should be referred or discussed early with units who have expertise in EPS surgery. Surgery should be performed by teams experienced in EPS surgery (GRADE 1B).

##### Guideline 7.2.2 – PD: Encapsulating peritoneal sclerosis: Management

We recommend that patients with EPS should have early dietetic referral and monitoring of nutritional status, with nutritional support by oral enteral, or often parenteral supplementation usually required (GRADE 1C).

##### Guideline 7.2.3 – PD: Encapsulating peritoneal sclerosis: Management

We suggest that there is no clear evidence to support a recommendation for the use of any medical therapy for treating EPS. Corticosteroids, immunosuppressants and tamoxifen have been used, and may be tried at the physician’s discretion (GRADE 2C).

##### Guideline 7.2.4 – PD: Encapsulating peritoneal sclerosis: Management

We suggest that PD should usually be discontinued after diagnosis of EPS with transfer to haemodialysis. However, this should be an individual patient decision considering, patient wishes, life expectancy and quality of life (GRADE 2C).

#### Guideline 7.3 – PD: Encapsulating peritoneal sclerosis

##### Guideline 7.3 1– PD: Encapsulating peritoneal sclerosis: Duration of PD therapy

We recommend that there is no optimal duration of peritoneal dialysis or indication for routine elective modality switching. Decisions regarding the duration of therapy should be tailored to the individual patient, taking into account clinical and social factors and patient wishes, and should follow the principles outlined in the ISPD Length of Time on Peritoneal Dialysis and Encapsulating Peritoneal Sclerosis Position Paper (GRADE 1C).

## Summary of audit measures for peritoneal dialysis

- **Audit Measure 1:** Availability of modality choice
- **Audit Measure 2:** Monitoring of modality switching
- **Audit Measure 3:** Patient to peritoneal dialysis nursing staff ratio
- **Audit Measure 4:** Availability of assisted PD, utilisation and outcomes
- **Audit Measure 5:** Systems in place to check medical equipment
- **Audit Measure 6:** Use of non-standard systems with documentation of clinical indication
- **Audit Measure 7:** Use of biocompatible solutions and indication for use
- **Audit Measure 8:** Audit of care pathway for dialysis preparation to include information given (including proportion of patients offered PD), when and who delivers it
- **Audit Measure 9:** Audit of information on modality options provided to patients presenting who urgently require RRT, and both initial and subsequent modality of RRT selected by these patients.
- **Audit Measure 10:** Audit of care pathway for catheter insertion to include timeliness and need for temporary haemodialysis
- **Audit Measure 11:** Catheter complications and their resolution
- **Audit Measure 12:** Frequency of solute clearance (residual and peritoneal) estimation
- **Audit Measure 13:** Cumulative frequency curves for the total solute clearance
- **Audit Measure 14:** Frequency of measurement of membrane function, residual urine and peritoneal ultrafiltration volume
- **Audit Measure 15:** Identify patients with fluid reabsorption in long dwell
- **Audit Measure 16:** Number of patients regularly requiring hypertonic (3.86% glucose) exchanges to maintain fluid balance
- **Audit Measure 17:** Identify anuric patients with a total fluid removal <750 ml per day.
- **Audit Measure 18:** Routine annual audit of infection prevention strategies
- **Audit Measure 19:** Routine annual audit of PD peritonitis rates (including proportion of culture negative cases)
- **Audit Measure 20:** Routine annual audit of infection outcomes
- **Audit Measure 21:** Cumulative frequency curves of plasma bicarbonate
- **Audit Measure 22:** Processes in place to increase awareness of interference of assays by icodextrin metabolites
- **Audit Measure 23:** Number of patients with diagnosis of EPS who are referred to designated specialist EPS centres.

## Rationale for clinical practice guidelines for peritoneal dialysis

### Peritoneal dialysis (PD) (guidelines PD 1.1–1.5)

#### Guideline 1.1 – PD: Equipment and resources

We recommend that Peritoneal Dialysis should be delivered in the context of a comprehensive and integrated service for renal replacement therapies, including haemodialysis (including temporary backup facilities), transplantation and conservative care. Both continuous ambulatory peritoneal dialysis (CAPD) and automated peritoneal dialysis (APD), in all its forms should be available (1C).

#### Guideline 1.1.2 – PD: Equipment and resources

We recommend that a dedicated PD nursing team should be part of the multidisciplinary team (1C).

#### Guideline 1.1.3 – PD: Equipment and resources

We recommend that where feasible, each unit has a designated lead clinician for PD (1C).

#### Guideline 1.1.4 – PD: Equipment and resources

We recommend that assisted PD should be available to patients wishing to have home dialysis treatment but unable to perform self-care PD, including as a temporary measure where a patient who is, or will become, independent is unable to perform PD alone (1C).

### Rationale

Evidence from observational studies or registry data, with all its limitations, indicate that peritoneal dialysis (PD) used in the context of an integrated dialysis programme is associated with good clinical outcomes, certainly comparable to haemodialysis in the medium term (HD) and potentially better in the first 2 years of dialysis [1–10]. NICE recommends PD as the initial dialysis treatment of choice of chronic kidney disease stage 5 for children aged 2 years or older, people with residual renal function and adults without significant associated comorbidities (NICE Clinical Guideline 125, 2011). The only randomised study (NECOSAD), comparing HD to PD as a first treatment showed no differences in 2 year quality adjusted life years or 5 year mortality, but the number randomised was insufficient to generalize this observation; notably, most patients in this national study had sufficient life-style preferences related to one modality to decline randomisation [11]. PD has a significant technique failure rate however, so patients need to be able to switch treatment modality (to either temporary or permanent HD) in a timely manner, which has implications for HD capacity and the timing for HD access creation.

PD modalities (CAPD v. APD) have a different impact on life-style; one randomised study found that APD creates more time for the patient to spend with family or continue employment but is associated with reduced quality of sleep [12]. APD is usually the preferred modality for children [13]. There are medical indications for APD (see sections 2, 3 and 4), but generally initial modality choice is a lifestyle issue. Studies suggest no difference in outcomes resulting from selection of CAPD or APD as initial PD modality [14–16].

The success of a PD programme is dependent upon specialised nurses with appropriate skills in assessing and training patients for PD, monitoring of treatment and with sufficient resources to provide continued care in the community. A randomised trial of more intensive training has shown that this reduces peritonitis risk [17] and there is some evidence to support the benefit of regular home reviews of PD technique [18] (see section 5). Several studies have documented the benefits of home visits in identifying new problems, reducing peritonitis and non-compliance [19–21]. The National Renal Workforce Planning Group, (2002), recommended a caseload of up to 20 PD patients per nurse. It is important to note that this was a minimum recommendation. For smaller adult units, and paediatric units, a significantly greater number of nurses than determined by this ratio will be required to maintain a critical number to provide adequate specialist nurse cover across the year and to cover periods of absence. This is increasingly relevant now with the decline in PD patient numbers and unit sizes that has occurred since the publication of the Workforce Planning document. It is also of note that the responsibilities of PD nurses vary significantly between units, for example in some additionally being responsible for inpatient PD care, such that the required staffing level will be higher than this minimum. Greater numbers of nurses will be required where assisted PD is performed by staff from the PD unit rather than other external organisations. The requirement for specialist nurses with the skills to deal with complex patient educational issues is highlighted by the ISPD Guideline (2016) for teaching PD to patients and caregivers [22]. Having a designated lead clinician for PD in each unit may help to promote PD as a therapy option and to develop clinical management policies.

Assisted PD, with provision of nursing support in the community to help with part of the workload and procedures associated with PD, is a useful option to overcome an important barrier to home dialysis therapy [23]. Assisted APD should be available for patients, who are often but not always elderly, wishing to have dialysis at home, but are unable to perform self-care PD [24] and may also be used as a temporary measure for established patients temporarily unable to perform PD independently or for those unable to start PD alone but may later become independent. Assisted PD provides at least equivalent outcomes to in-centre haemodialysis for older patients [25–27], and higher treatment satisfaction [27] and is a viable option for expanding home care in more dependent patients [25, 26].

- **Audit Measure 1:** Availability of modality choice
- **Audit Measure 2:** Monitoring of modality switching
- **Audit Measure 3:** Patient to peritoneal dialysis nursing staff ratio
- **Audit Measure 4:** Availability of assisted PD, utilisation and outcomes

#### Guideline 1.2 – PD: Equipment and resources

We recommend that all equipment and fluid used in the delivery and monitoring of PD therapies should comply with the relevant standards for medical fluids and devices [1].

- **Audit Measure 5:** Systems in place to check medical equipment

This is a legal requirement

#### Guideline 1.3 – PD: Equipment and resources

We recommend that the use of disconnect systems should be standard unless clinically contraindicated (1A)

- **Audit Measure 6:** Use of non-standard systems with documentation of clinical indication

### Rationale

Disconnect systems have been shown through randomised trials to be associated with a lower peritonitis risk, especially in infections due to touch contamination [28].

#### Guideline 1.4 – PD: Equipment and resources

We suggest that biocompatible PD solutions (solutions that have normal pH and/or low concentrations of glucose degradation products) should be used in patients experiencing infusion pain (2B).

#### Guideline 1.5 – PD: Equipment and resources

We suggest that biocompatible PD solutions (normal pH and/or low concentrations of glucose degradation products) may be considered for better preservation of residual renal function with long term (>12 month) use (2B).

- **Audit Measure 7:** Use of biocompatible solutions and indication for use

### Rationale

A minority of patients commencing PD will experience infusion pain, often severe enough to consider discontinuing the therapy. A double blind randomised study demonstrated that pain could be prevented by using a normal pH, bicarbonate-lactate buffered dialysis fluid (Physioneal) [29]. Subsequent clinical experience has found that the benefit of this more biocompatible solution on infusion pain results in immediate and sustained benefit, and is probably applicable to other biocompatible solutions.

The evidence of other forms of clinical benefit from the routine use of biocompatible solutions is more controversial. Standard solutions are clearly bio-incompatible, with low pH (~5.2), lactate rather than bicarbonate buffer, high osmolality and high concentrations of glucose which also result in high concentrations of glucose degradation products (GDPs). Many in vitro and ex vivo studies have demonstrated the relative toxicity of these solutions, with all of the bioincompatible features playing their part [30–35]. There is also strong observational evidence that firstly detrimental functional changes to the peritoneal membrane occur with time on treatment, which are more exaggerated in patients using solutions with high glucose concentration early in their time on therapy [36, 37] and secondly, that morphological changes occur that are related to time on treatment which include membrane thickening and vascular scarring [38]. Time on treatment is also the greatest risk factor for encapsulating peritoneal sclerosis (EPS) [39, 40].

These observations have led dialysis companies to develop and market ‘biocompatible’ solutions, with normalization of pH, and/or reduction of GDPs and variable approaches to buffering. In randomised clinical trials these solutions have been shown to improve the dialysate concentrations of biomarkers considered to be indicators of mesothelial cell and possibly membrane health [41–44]. Systemic benefits possibly include reduced circulating advanced glycation end-products [44] and better glycaemic control in diabetics [45]. Data is currently lacking on hard clinical endpoints including technique failure or patient survival. One non-randomised, retrospective observational study has found an improved patient but not technique survival; patients in this study using biocompatible solutions were younger, suggesting a selection bias that may not be fully adjusted for, so caution should be exercised in the interpretation of this study [46]. Similar findings have been reported in a subsequent observational study, which has the advantage of including analysis of cohorts matched for factors including cardiovascular comorbidity, socioeconomic status and centre experience [47].

However, the limitations of being a non-randomised study with no fixed indication for prescription of biocompatible fluid, with potential for selection bias, and with differences in characteristics of the unmatched groups still apply [47]. Non-randomised, observational studies have also suggested a beneficial effect of biocompatible solutions on peritonitis rates [48, 49], but the strength of the conclusions are limited by the non-randomised study design and possibility of other factors contributing to observed differences in infection rates. A secondary outcome of the randomised balANZ trial was of a reduction in peritonitis rates in group receiving biocompatible PD fluid [50]. However, the most recent and largest registry study reported an increased risk of peritonitis with biocompatible fluids [51] and a recent systematic review has not demonstrated a benefit of low-GDP biocompatible solutions on peritonitis rates, patient or technique survival [52]. Thus further studies are required to answer the question regarding the potential effect of biocompatible fluids on PD peritonitis. The balANZ study also demonstrated interesting differences in effect on peritoneal membrane function. The biocompatible fluid group had a higher initial transport state one month after starting the trial, but transport status was then stable, unlike the standard fluid group where transport sate increased progressively [53]. The impact of this effect on outcomes including technique survival warrants further study.

The area with the strongest evidence for clinical benefit of biocompatible solutions is in the preservation of residual renal function. Several studies have suggested a benefit of low-GDP biocompatible fluids on residual function, with the largest being the balANZ trial [54]. Whilst differences in ultrafiltration between groups (which may indirectly affect residual urine via effects on hydration), make interpretation of the actual effect of the fluids on residual renal function more difficult in some studies [55], three systematic reviews of existing trials demonstrate a benefit of biocompatible solutions on residual renal function, when used for more than 12 months [52, 56, 57]. We suggest that biocompatible solutions be considered for preservation of residual kidney function. Currently there is insufficient evidence to recommend that all patients should be treated with biocompatible solutions, especially as this may have a significant cost implication. The argument for their use may be stronger if there was not an economic disadvantage. However, we note that routine clinical practice in UK is for children receiving PD to routinely be treated with biocompatible solutions.

### Peritoneal dialysis (PD) (guidelines PD 2.1–2.4)

#### Guideline 2.1 – PD: Preparation for peritoneal dialysis

We recommend that all patients (and parents of paediatric patients) should, where possible, be adequately prepared for renal replacement therapy and this should include receiving information and education about PD treatment, delivered by an experienced member of the MDT. Patients commencing RRT in an unplanned fashion for whatever reason should receive this information once appropriate (1C). Fast track education and urgent PD catheter insertion with acute start of PD should be available, and be offered to suitable patients urgently starting on RRT who wish to avoid temporary haemodialysis, with the associated negative aspects of temporary vascular access and disruption to their lives (1C).

- **Audit Measure 8:** Audit of care pathway for dialysis preparation to include information given (including proportion of patients offered PD), when and who delivers it.
- **Audit Measure 9:** Audit of information on modality options provided to patients presenting who urgently require RRT, and both initial and subsequent modality of RRT selected by these patients.

### Rationale

The arguments and rationale for this guideline relate to the National Service Framework for Renal Services, Part 1. The reader is referred to standard 2, Preparation and Choice pp. 21–23. The vast majority of patients commencing dialysis are medically suitable to receive PD if they select it. Some commonly perceived medical “contraindications” to PD are overstated. The majority of patients with a previous history of major abdominal surgery may successfully be treated with PD [58]. It is also unusual to be unable to achieve target small solute clearances in the majority of larger patients (with the availability of APD, even when anuric).

When patients present needing prompt, unplanned start to renal replacement therapy, rapid insertion of a PD catheter with acute start of PD, along with fast track education regarding dialysis modalities, may allow a proportion to commence directly on PD, avoiding temporary vascular access and urgent haemodialysis [59–61]. Such patients who initially receive acute start of haemodialysis should receive follow up education regarding RRT options.

#### Guideline 2.2 – PD: Preparation for peritoneal dialysis

We recommend that, where possible, timing of PD catheter insertion should be planned to accommodate patient convenience, commencement of training between 10 days and 6 weeks and before RRT is essential to enable correction of early catheter-related problems without the need for temporary haemodialysis (1C).

- **Audit Measure 10:** Audit of care pathway for catheter insertion to include timeliness and need for temporary haemodialysis

### Rationale

The arguments and rationale for this guideline relate to the National Service Framework for Renal Services, Part 1. The reader is referred to standard 3, Elective Dialysis Access Surgery, pp. 24–26. The Moncrief catheter is buried subcutaneously and is designed to be left in this position, where it can remain for many months, until required [62].

#### Guideline 2.3 – PD: Preparation for peritoneal dialysis

We recommend that PD catheter insertion practice should be managed according to the Renal Association Peritoneal Access Guidelines. Paediatric PD access procedures will routinely be performed under general anaesthetic (Ungraded).

#### Guideline 2.4 – PD: Preparation for peritoneal dialysis

We recommend that peri-operative catheter care and catheter complications (leaks, hernias, obstruction) should be managed according to the International Society of Peritoneal Dialysis guidelines 2005, and for children, the European Elective Chronic Peritoneal Dialysis Guideline 2001 (Ungraded).

- **Audit Measure 11:** Catheter complications and their resolution

### Rationale

Recommendations for management of PD catheter insertion in adults are contained in the Renal Association Peritoneal Access Guidelines. The same principles apply in paediatric practice, except that procedures in children will routinely be performed under general anaesthetic. For management of the catheter in the peri-operative period, for catheter related problems including leak (internal and external), poor flow, obstruction and hernias, the guidelines developed by the International Society of Peritoneal Dialysis, www.ispd.org [63, 64] and the European Elective Chronic Peritoneal Guideline [13] should be used. Catheter problems due to increased intra-peritoneal pressure, especially leaks, hernias and prolapse are an important medical indication for the use of APD either temporarily or permanently; poor flow or catheter related flow pain should be treated with tidal APD. In the majority of cases where surgical repair for mechanical complications is required (e.g. catheter replacement, hernia repair) it is possible to avoid the need to temporary haemodialysis. In many PD patients, remaining residual renal function may permit an adequate period post-surgery before dialysis needs to be recommenced. Where PD does need to start soon after surgery, in many cases this may be safely achieved by initial use of APD with small volume exchanges and avoiding a day dwell in ambulant patients [65].

### Peritoneal dialysis (PD) (guidelines PD 3.1–3.3)

#### Guideline 3.1 – PD: Solute clearance

We recommend that both residual urine and peritoneal dialysis components of small solute clearance should be measured at least six monthly or more frequently if dependant on residual renal function to achieve clearance targets or if clinically or biochemically indicated in adults and in children. Both urea and/or creatinine clearances can be used to monitor dialysis adequacy and should be interpreted within the limits of the methods (1C).

- **Audit Measure 12:** Frequency of solute clearance (residual and peritoneal) estimation

### Rationale

Small solute clearance is one of the measurements of adequate dialysis treatment. Salt and water removal and acid-base balance are considered in sections 4 and 6 respectively. There are two issues in measuring small solute clearance that need to be taken into consideration.

First, the relationship to clinical outcomes of residual renal versus peritoneal small solute clearance is quantitatively different. Observational studies have shown that preserved renal clearance, in fact just urine volume, is associated with improved survival, independent of other known factors such as age and comorbidity [66, 67]. Randomised controlled trials designed to replace this residual renal function with peritoneal clearance did not show a proportional survival benefit [68, 69]. The recommendation to measure solute clearance six-monthly is driven primarily by the residual renal function component; indeed if dialysis dose has not been changed the peritoneal component will not be different and it would be acceptable just to measure the residual renal function. Indeed RRF can fall rapidly in some patients, certainly within a few weeks. If there are clinical concerns (e.g. if changes in symptoms, blood biochemistry, reported fall in urine output or after potential insults to residual renal function), or if achievement of solute clearance target is dependent on residual renal function, this should be undertaken more frequently.

Second, there are two potential surrogate solutes, urea and creatinine, that can be used to measure solute clearance in PD patients. There is no clear evidence as to which is the more useful clinically, and both have their problems. Current advice, therefore, is that either one or both can be used, ensuring that minimal clearances are achieved for at least one, but clinicians should be aware of their differing limitations. Urea clearances are limited by the difficulty in PD patients of estimating V accurately, whilst peritoneal creatinine clearances are affected by membrane transport characteristics (see Appendix).

#### Guideline 3.2.1 – PD: Solute clearance

We recommend that a combined urinary and peritoneal Kt/V_urea_ of 1.7/week or a creatinine clearance of 50 L/week/1.73m^2^ should be considered as minimal treatment doses for adults (1A). We recommend/suggest that clearance targets for children should be a minimum of those for adults (1C).

#### Guideline 3.2.2 – PD: Solute clearance

We recommend that the dose of dialysis should be increased in patients experiencing uraemic symptoms, or inadequate growth in children, even if meeting minimum clearance targets (1B).

#### Guideline 3.3 – PD: Solute clearance

We recommend that a continuous 24 h PD regime is preferred to an intermittent regime for anuric patients (1B).

- **Audit Measure 13:** Cumulative frequency curves for the total solute clearance

### Rationale

Two randomised controlled trials (ADEMEX and Hong Kong) have evaluated the impact of peritoneal solute clearances on clinical endpoints [68, 69]. Neither found that an increase of peritoneal Kt/V_urea_ > 1.7 was associated with an improvement in survival. Only one of these studies (ADEMEX) measured creatinine clearance, which was the solute used to make decisions in this case; patients in the control group achieved an average peritoneal creatinine clearance of 46 L/1.73m^2^/week and a total (urine plus renal) of 54 L/1.73m^2^/week. In setting a recommendation for minimal peritoneal clearances, to be achieved in anuric patients, the previous Renal Association guideline of Kt/V > 1.7 and creatinine clearance >50 L/1.73m^2^/week is supported by both the randomised and observational data. In the Hong Kong study, patients randomised to a Kt/V < 1.7, whilst their mortality was not significantly worse they had a significantly higher drop out rate, more clinical complications and worse anaemia. One observational longitudinal study demonstrated that patients develop malnutrition once the Kt/V falls below 1.7 with a three-fold increase in the death rate [70]. The NECOSAD study found that a creatinine clearance of <40 L/week or a Kt/V urea <1.5 was associated with increased mortality in anuric patients [71].

The vast majority of PD patients will be able to reach these clearance targets, especially if APD is employed [72]. These guidelines must however be viewed as recommendations for *minimal* overall clearance. In patients with residual renal function this renal clearance can be subtracted from the peritoneal clearance with confidence that the value of equivalent renal clearances is greater. Equally, in a patient achieving these clearances but experiencing uraemic symptoms, including reduced appetite or nutritional decline, or failing to achieve adequate acid base balance (see section 6) then the dialysis dose should be increased. Drop out due to uraemia or death associated with hyperkalaemia and acidosis was significantly more common in the control patients in the ADEMEX study [68]. In patients with borderline clearances, who fail to achieve these clearance targets, other aspects of patient wellbeing, long-term prognosis from other comorbidity and patient perspective should be considered in deciding whether switch of modality to haemodialysis is appropriate. It is important to note that spuriously low Kt/V urea may arise due to overestimation of V in patients with significant obesity (see Appendix).

ADEMEX randomised patients between a “standard” CAPD regime of 4 × 2 l exchanges (rather than a specific clearance value) vs enhanced prescription to obtain specified clearance targets [68]. Thus this study should not be used to justify routine reduction of dialysis prescription down to minimum clearance targets. The large ANZDATA observational study suggested a lower survival with low peritoneal Kt/V [73]. One possible interpretation of the data is that low peritoneal clearances were linked to reduced dialysis prescription in patients with good residual renal function.

There is a discrepancy between clearance of small solutes and larger molecules, which are more dependent on time of contact of dialysate with the peritoneal membrane than dialysate volume [74]. Thus continuous regimes are preferred to those with “dry” periods (e.g. NIPD), particularly in anuric patients, even if small solute clearance targets can be achieved without continuous therapy. An exception to this is in the situation where a patient still has a large residual renal function.

In paediatrics there is a lack of high quality evidence to determine clearance targets for children on PD. In small children and infants, Kt/V is likely to be disproportionately high compared with creatinine clearance and adult targets are particularly inadequate in these patients [75]. It is suggested by British Association of Paediatric Nephrology that the adult targets should be considered as minimum desirable, with an increase in PD prescription in the presence of features of uraemia, including inadequate growth [76]. Evidence in small numbers of subjects has suggested that in children increasing dialysis prescription may reach a point of no further benefit or adverse effects on nutrition due to increased dialysate protein removal [77].

### Peritoneal dialysis (PD) (guidelines PD 4.1–4.5)

#### Guideline 4.1 – PD: Ultrafiltration and fluid management

We recommend that peritoneal membrane function should be monitored regularly (6 weeks after commencing treatment and at least annually or when clinically indicated) using a peritoneal equilibration test (PET) or equivalent. Daily urine and peritoneal ultrafiltration volumes, with appropriate correction for overfill, should be monitored at least six-monthly (1C).

- **Audit Measure 14:** Frequency of measurement of membrane function, residual urine and peritoneal ultrafiltration volume

### Rationale

Assessment of membrane function, specifically solute transport rate and ultrafiltration capacity) is fundamental to PD prescription. (See Appendix for methodological description of membrane function tests). This is for the following reasons:

- There is considerable between-patient variability in both solute transport and ultrafiltration capacity that translates into real differences in achieved solute clearance and ultrafiltration unless they are accounted for in prescription practice [78–81]
- Membrane function is an independent predictor of patient survival; specifically high solute transport and low ultrafiltration capacity are associated with worse outcomes [82–86]
- Membrane function changes with time on therapy. There are early changes – usually during the first few weeks of treatment that can be avoided by performing tests 6 weeks after commencing PD. Later changes vary between patients but tend to be increasing solute transport and reduced ultrafiltration capacity; the rate of membrane change is accelerated in patients with earlier loss of residual renal function and greater requirement for hypertonic glucose solutions [87, 88].

The European Renal Best Practice advisory board have produced detailed recommendations for the methodology of evaluation of peritoneal membrane function in clinical practice, and for utilising the results in PD prescription [89].

Residual renal function, as discussed above, is one of the most important factors, along with age, comorbidity, nutritional status, plasma albumin and membrane function that predict survival in PD patients. Its rate of loss is variable and clinically significant changes can occur within 6 months. Total fluid removal is associated with patient survival, especially once anuric [85, 90, 91].

#### Guideline 4.2 – PD: Ultrafiltration and fluid management

We recommend that dialysis regimens resulting in fluid reabsorption should be avoided. Patients with high or high average solute transport, at greatest risk of this problem, should be considered for APD and icodextrin (1A).

- **Audit Measure 15:** Identify patients with fluid reabsorption in long dwell

### Rationale

Increased solute transport has been repeatedly shown to be associated with worse survival, especially in CAPD patients [82–84, 86]. The explanation for this association is most likely to be because of its effect on ultrafiltration when this is achieved with an osmotic gradient (using glucose or amino-acid dialysis fluids). The reason is twofold: first, due to more rapid absorption of glucose, the osmotic gradient is lost earlier in the cycle resulting in reduced ultrafiltration capacity. Second, once the osmotic gradient is dissipated the rate of fluid reabsorption in high transport patients is more rapid. This will result in significant fluid absorption, contributing to a positive fluid balance, during the long exchange.

These problems associated with high transport can be avoided by using APD to shorten dwell length and by using icodextrin for the long exchange to prevent fluid reabsorption. Several randomised controlled trials have shown that icodextrin can achieve sustained ultrafiltration in the long dwell [92–96] and that this translates into a reduction in extracellular fluid volume [97, 98]. Observational studies indicate that high solute transport is not associated with increased mortality or technique failure in APD patients, especially when there is also a high use of icodextrin [84, 85, 99]. Results from the ANZDATA Registry show that in high transport patients, treatment with APD was associated with a superior patient survival compared with CAPD [100]. Survival in low transport patients in contrast was lower with APD. A Korean registry study reported a benefit of icodextrin on patient and PD technique survival [101] but adequately powered randomised trials to confirm this are still needed [102].

A difference in practice for paediatrics is that patients with an underlying diagnosis of renal dysplasia are often polyuric, and so not so dependent on peritoneal ultrafiltration for maintenance of euvolaemia.

#### Guideline 4.3 – PD: Ultrafiltration and fluid management

We recommend that dialysis regimens resulting in routine utilisation of hypertonic (3.86%) glucose exchanges should be minimised. Where appropriate this should be achieved by using icodextrin or diuretics (1B).

- **Audit Measure 16:** Number of patients regularly requiring hypertonic (3.86% glucose) exchanges to maintain fluid balance

### Rationale

There is growing evidence that regular use of hypertonic glucose dialysis fluid (3.86%), and where possible glucose 2.27%, is to be avoided as far as possible. It is associated with acceleration in the detrimental changes in membrane function that occur with time on treatment [80, 103], as well as several undesirable systemic effects including weight gain [94, 104], poor diabetic control, delayed gastric emptying [105], hyperinsulinaemia [106] and adverse haemodynamic effects [107]. In addition to patient education to avoid excessive salt and fluid intake, where possible the use of hypertonic glucose should be minimised by enhancing residual diureses with the use of diuretics (e.g. frusemide 250 mg daily) [108]. Substituting icodextrin for glucose solutions during the long exchange will result in equivalent ultrafiltration whilst avoiding the systemic effects of the glucose load [94, 98, 107]. Observational evidence would suggest that icodextrin is associated with less functional deterioration in the membrane in APD patients [103].

#### Guideline 4.4 – PD: Ultrafiltration and fluid management

We recommend that treatment strategies that favour preservation of renal function or volume should be adopted where possible. These include the use of ACEi, ARBs (in adults only) and diuretics, and the avoidance of episodes of dehydration (1B).

### Rationale

This is the single most important parameter in PD patients, and also the one most likely to change with time. Clinically significant changes can occur within three months. Because secretion of creatinine by the kidney at low levels of function overestimates residual creatinine clearance, it is recommended to express this as the *mean* of the urea and creatinine clearances. Observational and randomised studies have shown that episodes of volume depletion, whether unintentional or in response to active fluid removal with the intent of changing blood pressure or fluid status, are associated with increased risk of loss in residual renal function [97, 98, 109]. Care should be taken not to volume deplete a PD patient too rapidly or excessively. The need to determine an appropriate target weight to avoid the cardiac complications of occult fluid overload, whilst avoiding loss of residual renal function due to excessive fluid removal is a major challenge in the management of the PD patient who has still has a significant residual urine output. The use of diuretics to maintain urine volume is not associated with a risk to renal clearances [108]. ACE inhibitors, (Ramipril 5 mg) [110] and ARBs (valsartan) [111] have been shown in randomised studies in adults to maintain residual diuresis. A Cochrane review also suggested superior preservation of residual function in PD with ACEis or ARBs [112]. Evidence for a benefit of ACE inhibitors or ARBs to preserve residual renal function in children is lacking, and a recent report from the International Pediatric Peritoneal Dialysis Network registry suggested that renin-angiotensin blockade could be associated with an increased risk of loss of residual renal function in children [113], and so these drugs are not recommended for preservation of kidney function in paediatric PD patients. Paediatric practice may also differ with the management of a subgroup of patients with renal dysplasia and a tendency to polyuria.

#### Guideline 4.5 – PD: Ultrafiltration and fluid management

We recommend that anuric patients who are overhydrated and consistently achieve a daily ultrafiltration of less than 750 ml in adults (or equivalent volume for body size in paediatrics) should be closely monitored. These patients may benefit from prescription changes and/or modality switch (1B).

- **Audit Measure 17:** Identify anuric patients with a total fluid removal <750 ml per day.

### Rationale

Observational studies have consistently shown that reduced peritoneal ultrafiltration is associated with worse survival rates; whilst this is seen in studies with or without residual urine [90], this effect is most marked in anuric patients [85]. In the only prospective study to have pre-set an ultrafiltration target (750 ml/day), patients who remained below this had higher mortality after correcting for age, time on dialysis, comorbidity and nutritional status. It is likely this association is multifactorial, but failure to prescribe sufficient glucose or icodextrin and a lower ultrafiltration capacity of the membrane were factors in this study and should be considered [85, 114]. The European guidelines have suggested a 1 l minimal daily ultrafiltration target [115] but there is insufficient evidence to say that such a target must be met at this stage. It is possible that in some patients with low ultrafiltration, this is appropriate to their low fluid intake, and that in these cases decreased survival possibly results from poor nutrition rather than fluid excess, and that increasing ultrafiltration would simply result in dehydration with its adverse effects. Blood pressure, salt (and fluid) intake, nutritional and fluid status, and presence of any features of uraemia should be taken into account. Nevertheless patients with less than 750 ml ultrafiltration once anuric should be very closely monitored and the potential benefits of modality switch considered.

### Peritoneal dialysis (PD) (guidelines PD 5.1–5.2)

#### Guideline 5.1 – PD: Infectious complications

##### Guideline 5.1.1 – PD infectious complications: Prevention strategies

We recommend that PD units should undertake regular audit of their peritonitis and exit-site infection rates, including causative organism, treatment and outcomes. They should enter into active dialogue with their microbiology department and infection control team to develop optimal local treatment and prevention protocols (1B).

##### Guideline 5.1.2 – PD infectious complications: Prevention strategies

We recommend that flush-before-fill dialysis delivery systems should be used for CAPD (1A).

##### Guideline 5.1.3 – PD infectious complications: Prevention strategies

We recommend that patients (and/or carers or parents) should undergo regular revision of their technique (at least annually or more frequently if indicated, such as after an episode of PD-related infection or a significant interruption to the patient performing PD) and receive intensified training if this is below standard (1C).

##### Guideline 5.1.4 – PD infectious complications: Prevention strategies

We recommend that initial catheter insertion should be accompanied by antibiotic prophylaxis (1B).

##### Guideline 5.1.5 – PD infectious complications: Prevention strategies

We recommend that invasive procedures should be accompanied by antibiotic prophylaxis and emptying the abdomen of dialysis fluid for a period commensurate with the procedure (1C).

##### Guideline 5.1.6 – PD infectious complications: Prevention strategies

We recommend that topical antibiotic administration should be used to reduce the frequency of exit-site infection and peritonitis (1A).

- **Audit Measure 18:** Routine annual audit of infection prevention strategies
- **Audit Measure 19:** Routine annual audit of PD peritonitis rates (including proportion of culture negative cases)

### Rationale

The rationale underpinning the guidelines in this section is laid out in a series of documents published by the International Society of Peritoneal Dialysis, available on their web-site: www.ispd.org.

**Prevention strategies**: The ISPD 2016 PD-related infections guideline, the ISPD 2011 position statement on reducing the incidence of PD-related infections, 2017 ISPD catheter-related infection recommendations and the 2012 ISPD guideline for prevention and treatment of catheter-related infections and peritonitis in paediatric patients receiving PD [116–119] place increasing emphasis on prevention strategies. Regular audit is essential to this progress with a team approach to quality improvement [117] and the following standards should be considered as minimal:

1. Peritonitis rates of less than 0.5 episode per patient year in adults and children
2. A primary cure rate of >80%
3. A culture negative rate of <20%

Patient training to perform PD technique by experienced PD nurses trained to do this as part of a formalised training programme is essential in patients commencing PD [120]. Greater experience of nurses providing training is associated with greater time to initial episode of peritonitis [121]. It is recommended that review of patient PD technique is performed on a regular basis, at least annually, or more frequently if there is evidence of inadequate technique or development of PD –related infection, or a significant interruption in the performing PD e.g. after a significant period of hospitalisation). Approaches that have been shown to reduce infection rates in randomised studies include increased intensity of training, use of flush before fill systems, antibiotic prophylaxis to cover catheter insertion and prevention of exit-site infections [116, 117]. Several studies have addressed the latter issue; following demonstration that the risk of *Staph aureus* exit site infection (the organism most frequently responsible) is associated with pre-existing skin carriage, several randomised studies demonstrated that clinical exit-site infection and associated peritonitis could be reduced by either nasal or exit-site application of mupirocin. This has led to the practice of applying mupirocin to all patients [122, 123] and this approach should be discussed with the local microbiology and infection control team. A systematic review has confirmed the benefits of prophylactic mupirocin in preventing exit-site infections and *Staph aureus* peritonitis [124] A more recent study, comparing mupirocin with gentamicin cream, found that the latter prevented both *Staph aureus* and *Pseudomonas* exit-site infections and peritonitis episodes [125]. This approach should be considered in patients with a known history of *Pseudomonas* infections; again the policy should be discussed and agreed with the local microbiology team.

#### Guideline 5.2 – PD: Infectious complications

##### Guideline 5.2.1 – PD infectious complications: Treatment

We recommend that exit site infection is suggested by pain, swelling, crusting, erythema and serous discharge; purulent discharge always indicates infection. Swabs should be taken for culture and initial empiric therapy should be with oral antibiotics that will cover *S. aureus and P. aeruginosa* (1B)*.*

##### Guideline 5.2.2 – PD infectious complications: Treatment

We recommend that methicillin resistant organisms (MRSA) will require systemic treatment (e.g. vancomycin) and will need to comply with local infection control policies (1C).

##### Guideline 5.2.3 – PD infectious complications: Treatment

We recommend that initial treatment regimens for peritonitis should include cover for bacterial Gram positive and Gram negative organisms including *Pseudomonas species* until result of culture and antibiotic sensitivities are obtained (1C).

- **Audit Measure 20:** Routine annual audit of infection outcomes

### Rationale

The International Society of Peritoneal Dialysis (ISPD) has developed a simple scoring system for exit site signs and symptoms which is easy to use and gives guidance on when to treat immediately rather than waiting for a swab result. Purulent discharge is an absolute indicator for antibiotic treatment [126].

The ISPD has become less dogmatic about the initial choice of antibiotic treatment for peritonitis, provided that gram positive and negative infections are covered [116]. It is recognised that patterns of resistance vary considerably and thus a local policy must be developed. Studies do not currently demonstrate a favoured regime [127]. For exit site infections the presence of a tunnel infection should be recognised as it may require more aggressive management. We concur with the ISPD guidelines that suggest suitable antibiotic dosing regimens, including options for intermittent and continuous dosing of intraperitoneal antibiotics. We also note their comment that infections from Gram negative organisms are more likely to lead to refractory or recurrent peritonitis. A single study suggested that treating Gram negative peritonitis with 2 appropriate antibiotics might be associated with better outcomes. It is also important to be aware of the potential for impaired absorption of oral antibiotics in some situations, e.g. co-prescription of ciprofloxacin with some phosphate binders [128].

We would emphasise the ISPD guidelines that it is important that timely PD catheter removal is undertaken in refractory PD peritonitis [116]. PD catheter removal or swap is also required in refractory exit site infections, and may be required earlier where there is a Pseudomonas infection or associated tunnel infection, which can be confirmed by ultrasound imaging [126, 129].

There will be a lower threshold in paediatrics for admission for IV antibiotics (at least for first 48 h), especially in infants and small children where oral antibiotics commonly cause diarrhoea/feed intolerance.

### Peritoneal dialysis (PD) (guidelines PD 6.1–6.4)

#### Guideline 6.1 – PD: Metabolic factors

We recommend that standard strategies to optimise diabetic control should be used; these should be complemented by dialysis prescription regimens that minimise glucose, including glucose-free solutions (icodextrin and amino-acids), where possible (1B).

### Rationale

Glycaemic control can be made worse by glucose absorption across the peritoneal membrane. Dialysis regimens that incorporate less glucose and more glucose free (amino acid, icodextrin) solutions have been shown to improve glycaemic control [130, 131]. Diabetes is a rare cause of end-stage renal failure in paediatrics, but these principles would also apply to children on PD who have diabetes. The IMPENDIA-EDEN randomised controlled study compared all-glucose regimes with regimes including both icodextrin and amino acid PD dialysis fluids in diabetic patients on PD demonstrated a 0.5% reduction in glycated haemoglobin [131]. Serum triglyceride, very-low-density lipoprotein, and apolipoprotein B also improved. However it is important to note that the intervention group suffered an increase in adverse events and deaths, including events related to extracellular fluid expansion [131]. It is therefore critical that this approach with use of low-glucose solutions is accompanied by careful monitoring of hydration and is not at the expense of a decline in fluid management. It also should not be an alternative to appropriate use of hypoglycaemic drugs, and monitoring for hypoglycaemia is important in patients where dialysate glucose load is reduced.

Although there is no strong equivalent evidence in paediatrics, it is suggested that the principles of minimisation of peritoneal glucose exposure to avoid obesity and reduce the adverse effects on peritoneal membrane function should also apply to children.

#### Guideline 6.2 – PD: Metabolic factors

We recommend that plasma bicarbonate should be maintained within the normal range. This can be achieved in the vast majority of patients by adjusting the dialysis dose and/or dialysate buffer concentration (1B).

- **Audit measure 21:** Cumulative frequency curves of plasma bicarbonate

### Rationale

Two randomised controlled trials have suggested that clinical outcomes, including gaining lean body mass and reduced hospital admissions are achieved if the plasma bicarbonate is kept within the upper half of the normal range [132, 133]. Generally this can be achieved by using dialysis fluids with a 40 mmol buffer capacity (lactate or bicarbonate results in similar plasma bicarbonate levels [134] and ensuring that the dialysis dose is adequate (see section 3 (b), above) [135]. However, for solutions with a lower buffering capacity, when patients are switched from an all lactate (35 mmol/l) to a 25 mmol bicarbonate: 10 mmol lactate mix, there is a significant improvement in plasma bicarbonate (24.4 to 26.1 mmol/l), such that a higher proportion of patients will fall within the normal range [136]. Whilst bicarbonate solutions may have a role in biocompatibility (see section 1(e), above), they are generally not required to achieve satisfactory acid-base balance in adults. The main reason for using a 35 mmol buffer capacity solution (25:10 bicarbonate:lactate mix) is to avoid excessive alkalinisation [137]. Plasma bicarbonate will also be affected by some phosphate binders that either increase, or occasionally (sevelamer hydrochloride) decrease concentrations. In paediatric practice in UK, use of neutral pH/low GDP solutions is routine.

Control of acidosis is especially important in malnourished patients who may benefit from the glucose available in dialysis solutions as a calories source. Amino acid solutions were developed in an attempt to address protein calorie malnutrition and several randomised studies have been conducted. In using amino acid solutions it is essential to ensure that acidosis does not develop and to use the solution at the same time as there is a significant intake of carbohydrate [138]. Despite demonstration that amino acids delivered in dialysis fluids are incorporated into tissue protein, the randomised trials have failed to show benefit in terms of hard clinical endpoints [139, 140].

#### Guideline 6.3 – PD: Metabolic factors

We suggest that central obesity can worsen or develop in some PD patients. The risk of this problem, and associated metabolic complications, notably increased atherogenicity of lipid profiles and insulin resistance, can be reduced by avoiding excessive glucose prescription and using icodextrin (2C).

### Rationale

Weight gain, or regain, is common after starting peritoneal dialysis and this is associated with a worsening in the lipid profile [141, 142], though there may not be a significant difference from haemodialysis [143]. Randomised studies comparing glucose 2.27% with icodextrin in the long exchange have shown that the latter prevents weight gain, which in body composition studies is at least in part fat weight. Substituting icodextrin for 2.27% glucose in the long dwell also improves insulin resistance [144]. There is limited available trial data on the benefit of statins in PD patients with a hard clinical endpoint. The 4D and AURORA studies did not include PD patients, and whilst SHARP included 33% dialysis patients, only 5% of the study patients were receiving PD. There is no data on the effects of lipid-lowering in children on PD. There are good reasons for believing that the lipid abnormalities in the PD patient population may be different to patients on HD, and potentially more atherogenic. The KDIGO guideline for lipid management in CKD suggests that statins and/or ezetimibe are not commenced in dialysis patients, but that they are continued if a patient is receiving them before stating dialysis [145] though it is important to note that the majority of evidence this is based on is derived in haemodialysis patients. Observational data in one trial of adults has suggested a possible benefit of statins in adults receiving PD [146]. The Canadian Society of Nephrology Guidelines suggest that statins and/or ezetimibe should be considered for adult PD patients [147].

#### Guideline 6.4 – PD: Metabolic factors

We recommend that awareness of the effects of Icodextrin on assays for estimation of amylase and glucose (using glucose dehydrogenase) should be disseminated to patients, relatives, laboratory and clinical staff (1C).

- **Audit Measure 22:** Processes in place to increase awareness of interference of assays by icodextrin metabolites

### Rationale

Use of icodextrin is associated with circulating levels of metabolites that can interfere with laboratory assays for amylase (or actually suppress amylase activity) [148–151] and for glucose when finger-prick tests that utilise glucose dehydrogenase as their substrate are employed (manufactured by Boehringer Mannheim) [152–155]. In the case of amylase, the measured level will be reduced by 90%, leading to the potential failure in the diagnosis of pancreatitis. No adverse events have been reported, but clinicians should be aware of this possibility. If clinical concern remains then plasma lipase can be used. In the case of glucose measurements, the methods using glucose dehydrogenase will overestimate blood glucose levels, leading to a failure to diagnose hypoglycaemia. This has been reported on several occasions in the literature and has contributed to at least one death. Typically these errors occur in places and circumstances in which staff not familiar with peritoneal dialysis work, for example emergency rooms and non-renal wards. A number of solutions to this problem are under active review (e.g. use of alarm bracelets) but it is also the responsibility of health-care professionals to ensure that clinical environments in which their patients using icodextrin may find themselves are notified of this issue on a routine basis.

### Peritoneal dialysis (PD) (guidelines PD 7.1–7.3)

#### Guideline 7.1 – PD: Encapsulating peritoneal sclerosis

##### Guideline 7.1.1 – PD: Encapsulating peritoneal sclerosis: Diagnosis

We recommend that the diagnosis of encapsulating peritoneal sclerosis (EPS) requires the presence of a combination of clinical and radiological features of intestinal obstruction and encapsulation GRADE 1B.

##### Guideline 7.1.2 – PD: Encapsulating peritoneal sclerosis: Diagnosis

We recommend that the radiological technique of choice for the diagnosis of encapsulating peritoneal sclerosis (EPS) is CT scanning GRADE 1B.

##### Guideline 7.1.3 – PD: Encapsulating peritoneal sclerosis: Diagnosis

We recommend that radiological and biochemical screening methods are NOT of sufficient sensitivity and specificity to be used clinically to identify early or imminent development of EPS in asymptomatic PD patients (GRADE 1C).

### Rationale

Encapsulating peritoneal sclerosis (EPS) is rare, but serious complication of long-term PD. It involves formation of an inflammatory, and later fibrotic, “cocoon” surrounding the gastrointestinal tract [156]. This results in features of abdominal inflammation and intestinal obstruction. Symptoms may include abdominal pain, nausea, vomiting and haemoperitoneum and may predate definitive diagnosis by significant time periods in some instances. Typical appearances will be noted at laparotomy or laparoscopy. EPS should be distinguished from the thickening of the peritoneal membrane that typically occurs with time on PD, but which is not associated with obstructive features. Changes in peritoneal membrane small solute transport and ultrafiltration capacity often occur [157–159], but are also common in long-term PD and not always present in EPS, so are not of diagnostic value for EPS. There is no gold standard for the diagnosis of EPS, and it is recommended that the condition is diagnosed by the presence of the combination of characteristic clinical and radiological features [160, 161]. A challenge in this is that there is significant heterogeneity in the condition with variation of severity and extent of peritoneal involvement [156, 162, 163]. The epidemiology, clinical features, investigation and management of EPS in paediatric patients is similar to that in adults [164, 165]. Recommendations are developed from the UK Encapsulating Peritoneal Sclerosis Clinical Practice Guidelines 2009 [166].

Radiology plays a key role in the diagnosis of EPS. Plain abdominal X-rays may show features of bowel obstruction, but are non-diagnostic, except in cases where peritoneal calcification is present as a feature suggestive of EPS. CT scanning is recommended as the definitive radiological investigation for the diagnosis of EPS [167–172]. It has high reproducibility and evaluation has provided the basis of a standardised approach to CT diagnosis of EPS [171]. The presence of peritoneal calcification, bowel wall thickening, bowel tethering, and bowel dilatation are the features with greatest agreement between reporting radiologists [171]. Abdominal ultrasound may detect characteristic features in EPS [173]. However, there is a limitation to depth of penetration of sound waves which may limit ability for thorough evaluation of the abdomen, and it is operator-dependent. Small bowel contrast studies may also have a role in defining the presence of strictures prior to surgery.

At present, there are no investigations that can be recommended to monitor or screen patients on long-term PD to identify those who will develop EPS. One study has demonstrated that in patients developing EPS, who had abdominal CT scans for other reasons within a period of a year or less prior to diagnosis of EPS, there were no radiological abnormalities to suggest imminent development of EPS [174].

#### Guideline 7.2 – PD: Encapsulating peritoneal sclerosis

##### Guideline 7.2.1 – PD: Encapsulating peritoneal sclerosis: Management

We recommend that patients with suspected encapsulating peritoneal sclerosis (EPS) should be referred or discussed early with units who have expertise in EPS surgery. Surgery should be performed by teams experienced in EPS surgery (GRADE 1B).

##### Guideline 7.2.2 – PD: Encapsulating peritoneal sclerosis: Management

We recommend that patients with EPS should have early dietetic referral and monitoring of nutritional status, with nutritional support by oral enteral, or often parenteral supplementation usually required (GRADE 1C).

##### Guideline 7.2.3 – PD: Encapsulating peritoneal sclerosis: Management

We suggest that there is no clear evidence to support a recommendation for the use of any medical therapy for treating EPS. Corticosteroids, immunosuppressants and tamoxifen have been used, and may be tried at the physician’s discretion (GRADE 2C).

##### Guideline 7.2.4 – PD: Encapsulating peritoneal sclerosis: Management

We suggest that PD should usually be discontinued after diagnosis of EPS with transfer to haemodialysis. However, this should be an individual patient decision considering, patient wishes, life expectancy and quality of life (GRADE 2C).

- **Audit Measure 23:** Number of patients with diagnosis of EPS who are referred to designated specialist EPS centres.

### Rationale

EPS is a rare and complex condition, whose optimal management requires integrated care from an expert team experienced in managing this condition. Multiple disciplinary input includes PD physicians, nurses, surgeons, dieticians, radiologists and intensive care physicians.

There is increasingly strong evidence for a central role for surgery in the management of EPS [175–178]. Whilst earlier experience of EPS reported a high mortality for patients with this condition, and complications following surgery, in experienced hands, surgery results in high rates of resolution of symptoms and survival, and possibly superior relief of obstruction compared with conservative treatment with nutrition and/or drug treatment [178]. Surgery should be performed by a surgical team which has a high level of expertise and experience with EPS, and the appropriate multidisciplinary input and peri-operative renal and intensive care support. Surgical units at Manchester (Mr Titus Augustine), and Cambridge (Mr Chris Watson) are designated as national referral centres for surgery relating to EPS in England by NCG (National Commissioning Group). An early surgical opinion facilitates decisions regarding the need for, preparation and timing of surgery. Indications for surgery include non-responsiveness to medical treatment, bowel obstruction (acute and recurrent subacute), intraperitoneal bleeds, and peritonitis. A proportion of patients with EPS may have a good outcome without surgery so further work to define those most likely to benefit from surgery is needed. Where possible, surgery should be timed to take place electively before the patient is too ill or nutritionally depleted. Surgery involves careful dissection of thickened peritoneum from bowel loops to achieve maximal removal of sclerotic membrane from the bowel wall, whilst avoiding inadvertent perforation [176].

Reduced nutritional intake resulting from intestinal dysfunction, plus an ongoing inflammatory state in EPS, can lead to severe protein energy wasting [179, 180]. Nutritional state is associated with survival in EPS. Patients with EPS should be referred early to a renal dietician to allow nutritional assessment, monitoring and institution of nutritional support where needed. In more severe cases, parenteral nutrition may be required [180], and in patients where intestinal function does not recover, this may be required on a permanent basis. In milder cases, nutrition support may be managed with an energy dense diet or prescription of oral nutritional supplements and anti-emetics. Where patients are unable to tolerate adequate oral intake, nasogastric or nasojejunal feeding may be utilised.

Whilst there has been much interest in drug treatments for EPS, there is no robust evidence to support the use of anti-inflammatory or antifibrotic drugs in this condition. Corticosteroids have been most commonly used, particularly in the Japanese literature [178]. Any benefit is most likely with use in the early inflammatory stage of EPS. However there is not strong objective evidence for their effectiveness, and in EPS side effects of immunosuppression and protein catabolism are a particular concern. There are reports of use of immunosuppressants including azathioprine and cyclosporine in EPS. However evidence is largely as case reports, and as a common setting for development of EPS is following transplantation, in patients taking these drugs, their therapeutic effectiveness is doubtful. There is increasing interest in the role of tamoxifen, which is effective in other fibrotic conditions, in EPS [181, 182]. There is a suggestion from retrospective data of a beneficial effect of tamoxifen on survival [183] or that it could even have a preventative role [184], but robust evidence is currently lacking.

PD is usually discontinued and the PD catheter removed after diagnosis of EPS, with transfer to haemodialysis. However, as some cases are mild, the individual patient’s wishes and clinical state should be considered, as stopping PD may not be appropriate in a patient with mild symptoms and a poor long term prognosis, where continuation of PD and/or later conservative management may be appropriate. Also, there is experience in Japan of leaving the PD catheter in and performing peritoneal lavage after diagnosis of EPS, with observational non-randomised studies suggesting some benefit, though this approach is not widespread in other countries [185, 186].

#### Guideline 7.3 – PD: Encapsulating peritoneal sclerosis

##### Guideline 7.3 1– PD: Encapsulating peritoneal sclerosis: Duration of PD therapy

We recommend that there is no optimal duration of peritoneal dialysis or indication for routine elective modality switching. Decisions regarding the duration of therapy should be tailored to the individual patient, taking into account clinical and social factors and patient wishes, and should follow the principles outlined in the ISPD Length of Time on Peritoneal Dialysis and Encapsulating Peritoneal Sclerosis Position Paper (GRADE 1C).

### Rationale

The risk of developing EPS is extremely low in the first 3 years of PD and low before 5 years of therapy. The overall reported incidence typically varies between 0.5–3% in reported series [187–190]. Whilst the risk increases with time, the majority of patients on longer term PD will not develop EPS. The Scottish Renal Registry is notable in reporting a steeper rise of incidence with time on PD, with 8.1% risk of EPS after 4–5 years of PD [189]. Thus consideration of management of patients remaining on PD for longer term is warranted. However, it is unknown what impact elective discontinuation of PD after a certain period of time will have on the risk of developing EPS. A significant proportion of cases of EPS occur after discontinuing PD (either transplantation [191, 192] or switching to haemodialysis), so it is even possible that elective switching from PD could increase, rather than decrease, the risk of developing EPS. Discontinuing PD may also have potentially major adverse negative medical and social effects in some patients. Concern about EPS risk on long-term PD should be balanced against reports showing relatively good outcomes for EPS, relative to other competing risks [190], and good outcomes and success rates for EPS surgery when required. Thus routine discontinuation of PD after a fixed period of time cannot be recommended. The risks and benefits of continuing PD or dialysis modality change should be considered and discussed with the individual patient, as recommended in the ISPD 2009 Length of Time on Peritoneal Dialysis and Encapsulating Peritoneal Sclerosis Position Paper [193] (revised position paper will be published 2017).

## Lay summary

These guidelines cover all aspects of the care of patients who are treated with peritoneal dialysis. This includes equipment and resources, preparation for peritoneal dialysis, and adequacy of dialysis (both in terms of removing waste products and fluid), preventing and treating infections. There is also a section on diagnosis and treatment of encapsulating peritoneal sclerosis, a rare but serious complication of peritoneal dialysis where fibrotic (scar) tissue forms around the intestine. The guidelines include recommendations for infants and children, for whom peritoneal dialysis is recommended over haemodialysis.

Immediately after the introduction there is a statement of all the recommendations. These recommendations are written in a language that we think should be understandable by many patients, relatives, carers and other interested people. Consequently we have not reworded or restated them in this lay summary. They are graded 1 or 2 depending on the strength of the recommendation by the authors, and A-D depending on the quality of the evidence that the recommendation is based on.

## Appendix

### Assessment of membrane function in adult PD patients

- A number of methods to assess peritoneal membrane have been developed, the most commonly used, supported by clinical observation being the Peritoneal Equilibration Test (PET). This test measures two aspects of membrane function, low molecular weight solute transport (expressed as the dialysate:plasma ratio of creatinine at four hours), and the ultrafiltration capacity of the membrane. In the PET as originally described, ultrafiltration capacity is the net volume of ultrafiltration achieved at four hours using a 2.27% glucose exchange [194, 195]. In the simplified Standard Permeability Analysis (SPA) test, it is the net volume of ultrafiltration using a 3.86% exchange [196, 197].
- Using a standard PET, an ultrafiltration capacity of <200 mls (including overfill) is associated with a 50% risk of achieving <1000 mls ultrafiltration in anuric patients. Using a SPA test, an ultrafiltration capacity of <400 mls indicates ultrafiltration failure.
- The methods of performing PET and SPA tests are well described in the literature, The following points should be remembered in the interpretation of results:
  - High concentrations of glucose interfere with many assays for creatinine. It is important to work with the local biochemists to ensure that the appropriate correction for measurement of creatinine in dialysate has been taken into account.
  - Remember that dialysis bags are overfilled, mainly due to the additional fluid volume required to perform the ‘flush before fill’ procedure. Dialysis manufacturers are being encouraged to publish overfill volumes which differ significantly. The typical volume is 100-200 ml. The value of 200 ml UF capacity defining ultrafiltration failure quoted above *includes* the flush volume as this is easier for patients to perform (the alternative is weighing before and after flush which is time consuming and difficult).
  - The patient should follow their usual dialysate regime, draining out as completely as possible before the test dwell. Large residual volume of dialysate will affect the results.
  - Intra-patient variability of the ultrafiltration capacity (~ 20%) is greater than for the solute transport (<10%). Results of the PET/SPA, in particular the ultrafiltration capacity, should always be interpreted in the light of additional exchanges performed during the same 24–48 h period (usually collected to assess solute clearance – see below).
  - The PET/SPA are not surrogates for measuring solute clearance.
- The PET or SPA should be seen as a regular screening test to monitor membrane function and in most cases will explain clinically evident. Ultrafiltration problems. More detailed assessment of the membrane can be undertaken, in particular the double mini-PET. For further advice on this see the European Renal Best Practice Guidelines for assessing membrane function

### Measurement of solute clearance in adult PD patients

In measuring solute clearance and planning changes to the dialysis regime, three clinical parameters are essential: Estimates of (1) *patient size*, (2) peritoneal *solute transport* and (3) *RRF*. In each case, the choice of surrogate “toxin”, urea or creatinine, interacts with each of these parameters in different ways. At present, there is no clear evidence from the literature that one surrogate is superior to another. Where possible, clinicians should measure both, attempt to reach at least one of the targets, and understand why there appears to be a discrepancy. A number of commercial computer programs exist that are designed to aid dialysis prescription. Whilst some have been validated, good practice dictates that a change in dialysis prescription is checked for efficacy by repeating clearance studies.

#### Patient size

In calculating urea clearances, patient size is expressed as an estimate of the total body water (volume of distribution of urea). It is recommended that the Watson formula is used for this [196]:

$$ \mathrm{Males}:\mathrm{V}=2.447\hbox{--} {0.09156}^{\ast }\ \mathrm{age}\ \left(\mathrm{years}\right)+{0.1074}^{\ast }\ \mathrm{height}\ \left(\mathrm{cm}\right)+{0.3362}^{\ast }\ \mathrm{weight}\ \mathrm{kg}\Big) $$

$$ \mathrm{Females}:\mathrm{V}=-2.097+{0.1069}^{\ast }\ \mathrm{age}\ \left(\mathrm{years}\right)+{0.2466}^{\ast }\ \mathrm{weight}\ \left(\mathrm{kg}\right) $$

Anthropometric equations estimating TBW may produce results significantly different to gold standard dilution techniques (REF). This will impact on estimates of Kt/V and is of relevance if borderline Kt/V values are obtained [197, 198]. Alternatively 58% of body weight (kg) may be used; this is less precise, and will give lower values for Kt/V, especially in obese patients. Creatinine clearances should be corrected for body surface area, normalising to 1.73 m^2^.

#### Peritoneal solute transport

Solute transport rates have an important influence on peritoneal creatinine clearance, but not on urea clearance. This means that it is easier to achieve creatinine clearance targets in high transport patients. It should be remembered, however, that these patients might have less satisfactory ultrafiltration. In designing optimum dialysis regimens, patients with low solute transport will require equally spaced medium length dwells, such as are achieved with CAPD and single extra night exchanges (e.g. 5 × 2.5 l exchanges). Those with high transport are more like to achieve targets with short dwells (APD) plus polyglucose solutions (e.g. 4 × 2.5 l exchanges overnight, 1 × 2.5 l evening exchange and 1 × 2.5 l daytime icodextrin).

#### Residual renal function (RRF)

This is the single most important parameter in PD patients, and also the one most likely to change with time. Clinically significant changes can occur within three months. Because secretion of creatinine by the kidney at low levels of function overestimates residual creatinine clearance, it is recommended to express this as the mean of the urea and creatinine clearances.

### Estimating Total Ultrafiltration

The total achieved ultrafiltration is best measured from the 24-h dialysate collections used to calculate solute clearance. For APD patients this is simple as machines now calculate the ultrafiltration volumes precisely. Furthermore, many models store this information over several weeks so that an average value can be obtained. In CAPD patients it is important to remember that each bag is overfilled to achieve flush before fill; the total dialysate drain volume must be measured and sampled from to calculate solute clearance accurately, but the overfill must then be subtracted to calculate the net ultrafiltration. If this is not done then over a 24-h period the overestimate of ultrafiltration may be anything from 200 to 800 ml depending on manufacturer [199, 200].

Peritoneal sodium losses are largely determined by convection and are thus proportional to the ultrafiltration volume. Typically 1 l of ultrafiltration results in 100 mmol of sodium loss in CAPD patients and 70–80 mmol in APD patients.

### Assessment of membrane function in Paediatric PD patients estimating Total Ultrafiltration

Methodology for the measurement of peritoneal membrane function by PET and short PET in paediatric patients is described by Warady and Jennings [201].

### Measurement of solute clearance in Paediatric PD patients

Estimation of the volume of total body water for determination of V in Kt/V in children may be by the equations described by Morgenstern et al. [202].
